# Deep-Learning-Based Automated Classification of Chinese Speech Sound Disorders

**DOI:** 10.3390/children9070996

**Published:** 2022-07-01

**Authors:** Yao-Ming Kuo, Shanq-Jang Ruan, Yu-Chin Chen, Ya-Wen Tu

**Affiliations:** 1 Department of Electronic and Computer Engineering, National Taiwan University of Science and Technology, Taipei 106, Taiwan; m10902405@mail.ntust.edu.tw (Y.-M.K.); sjruan@mail.ntust.edu.tw (S.-J.R.); 2 Sijhih Cathay General Hospital, New Taipei 221, Taiwan; cgh398506@cgh.org.tw

**Keywords:** speech sound disorder, speech disfluency classification, Chinese speech sound disorder dataset, machine learning, artificial intelligence

## Abstract

This article describes a system for analyzing acoustic data to assist in the diagnosis and classification of children’s speech sound disorders (SSDs) using a computer. The analysis concentrated on identifying and categorizing four distinct types of Chinese SSDs. The study collected and generated a speech corpus containing 2540 stopping, backing, final consonant deletion process (FCDP), and affrication samples from 90 children aged 3–6 years with normal or pathological articulatory features. Each recording was accompanied by a detailed diagnostic annotation by two speech–language pathologists (SLPs). Classification of the speech samples was accomplished using three well-established neural network models for image classification. The feature maps were created using three sets of MFCC (Mel-frequency cepstral coefficients) parameters extracted from speech sounds and aggregated into a three-dimensional data structure as model input. We employed six techniques for data augmentation to augment the available dataset while avoiding overfitting. The experiments examine the usability of four different categories of Chinese phrases and characters. Experiments with different data subsets demonstrate the system’s ability to accurately detect the analyzed pronunciation disorders. The best multi-class classification using a single Chinese phrase achieves an accuracy of 74.4 percent.

## 1. Introduction

Speech sound disorders (SSDs) are one of the most common disorders in preschool and school-age children. Any issue or combination of difficulties with perception, motor production, or phonological representation of speech sounds and speech segments—including phonotactic rules controlling allowable speech sound sequences in a language is referred to as an SSD. According to a 2012 National Center for Health Statistics study [1], 48.1 percent of 3- to 10-year-old children and 24.4 percent of 11- to 17-year-old children with a communication impairment had just speech sound difficulties. Children with speech difficulties had a 76.6 percent use rate of speech intervention services, as reported by their parents [1]. Based on [2], speech delay or SSDs affect 2.3 percent to 24.6 percent of school-aged children.

There are two types of SSDs: organic and functional. An underlying motor/neurological, structural, or sensory/perceptual reason causes organic SSDs. There is no known cause for functional speech sound disorders; they are idiopathic. Functional SSDs are divided into two categories: motor production of speech and linguistic aspects of speech production. These issues have been referred to as articulation and phonological disorders, respectively, in the past. Errors (such as distortions and replacements) in producing particular speech sounds focus on articulation disorders. Phonological disorders are characterized by predictable, rule-based mistakes that influence several sounds (e.g., fronting, backing, and final consonant deletion) [3].

When a child has poor intelligibility, parents can visit a rehabilitation clinic and then be referred to SLPs for examination and training following assessment. According to [4], it takes an average of 54 min per case for assessment and analysis. Because there is a shortage of speech–language pathologists (SLPs) in Taiwan [5], children with SSDs often have to spend a longer waiting time visiting a clinic or the rehabilitation department of a medical institution. The waiting period is also a golden opportunity to miss out on treatment. Moreover, the lack of clarity in children’s speech can easily affect children’s social and communication interactions. Some children’s poor mastery of phonological rules can affect their future phonetic or intonation awareness [6]. According to the literature, speech therapy effectively improves children’s condition if started early [7]. The diagnosis of speech sounds varies depending on the method or location of the speech sound, so we can classify and model the features of the speech sound into specific categories. Correct diagnosis of pronunciations is the first step in clinical treatment, as the elicitation techniques vary by class. However, now in Taiwan, there is a lack of standardized assessment tools. The evaluation procedure may differ from one SLP to another due to differences in auditory awareness and not having a standard evaluation tool. Furthermore, as there are no normative models to compare evaluated instances to, it is difficult to make meaningful comparisons between them. Additionally, the assessment content varies from monotone vocabulary to spontaneous speech. The overall workflow is lengthy and laborious, and therapists are frequently required to complete the assessment and health education in less than 30 min, which is exhausting and inconvenient. Therefore, the availability of automatic classification assessment tools can save time for SLPs and quickly identify speech problems in children and provide accurate treatment directions.

### 1.1. Disorders Characterizations

The phonological processes are divided into syllabic structure, substitution, and assimilation. Substitution processes can be classified by their articulation method or location. The term “place of articulation” refers to the point at which two speech organs, such as the tongue and teeth, come into contact to produce speech sounds. The manner in which the articulatory structures are shaped and coordinated determines the manner in which they articulate, and common diagnoses such as stopping and affrication are extremely diverse. To create different speech sounds, we experimented with various airflow methods, the degree of airflow obstruction, and the duration of airflow. According to [8], the most common types of errors in preschool children are backing, stopping, affrication, and unaspiration. The current study focuses on four types of errors that are frequently encountered: stopping, backing, final consonant deletion process (FCDP), and affrication.

Using spectrograms to analyze speech problems can reveal a wealth of information that cannot be analyzed by the ear. The horizontal axis of the spectrogram is the time scale, and the vertical axis is the frequency of the sound. The vertical axis is the frequency of the sound, and from the bottom to the top is the logarithmic scale from 0 to 20,000 Hz, which represents the range of audible sound. Using a logarithmic scale emphasizes the range of frequencies emitted by the vocal cords. The spectrum’s brightness indicates the sound’s magnitude at the corresponding time and frequency. The higher the dB value, the brighter the color, and the lower the dB value, the darker the color.

#### 1.1.1. Stopping

**Figure 1:**
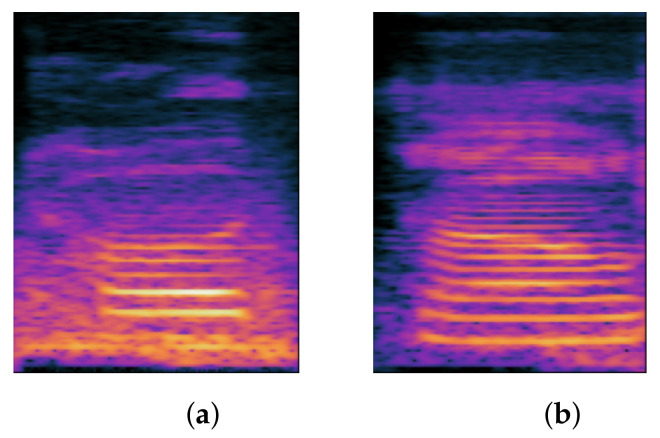
Spectrum comparison of stopping and correct: (**a**) stopping; (**b**) correct.

Stopping refers to when non-stop sounds are incorrectly pronounced as stop; in Chinese, stop sounds include ㄅ/p/, ㄆ/p^h^/, ㄉ/t/, ㄊ/t^h^/, ㄍ/k/, and ㄎ/k^h^/; therefore, when we mispronounce other sounds into the above six sounds in our daily lives, we will experience stopping. It is referred to as stopping, as in /k^h^u⌉⌋ tsɯ˧/ read as /tu⌉⌋ tsɯ˧/, but the stop sound contains the two sounds ㄍ/k/ and ㄎ/k^h^/. When we mispronounce the pronunciation as ㄍ/k/ or ㄎ/k^h^/ speech in clinical practice, we do not refer to it as stopping but rather as backing, as explained in the following subsection. When the sound spectrum is analyzed, we can see that the stop exhibits the following characteristics. The first is the duration of silence, which is the duration of the stop being blocked; The time interval between the burst and the beginning of the vowel is referred to as the voice onset time (VOT). We can distinguish various speech sounds based on the acoustic characteristics listed above. Figure 1 depicts the spectrogram difference between the stopping and normal pronunciation.

#### 1.1.2. Backing

**Figure 2:**
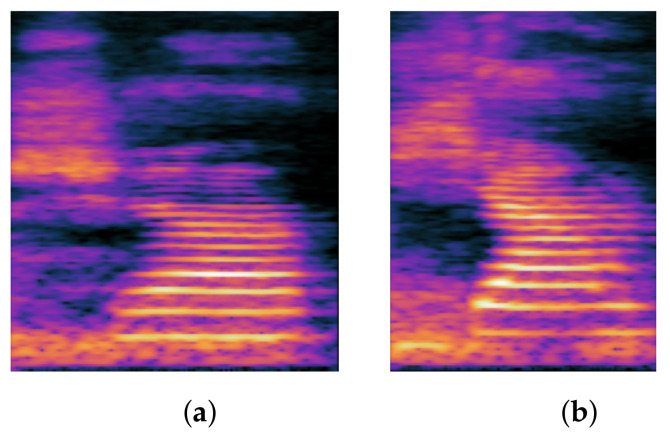
Spectrum comparison of backing and correct: (**a**) backing; (**b**) correct.

The Chinese backing consonants include ㄍ/k/, ㄎ/k^h^/ and ㄏ/x/.When we pronounce Chinese pronunciation, the stop, affrication, fricative, etc. are replaced by the ㄍ/k/ and ㄎ/k^h^/, and we call it backing. For example, /t^h^u⌉⌋ tsɯ˧/ becomes /k^h^u⌉⌋ tsɯ˧/. In English, backing can occur at any point in the word, but in Chinese, the phonological progression of backing occurs exclusively in consonants, and thus the error occurs at the beginning of the word, which is referred to as the initial consonant in Chinese. The term “backing” refers to a speech sound produced by the soft palate being held upward by the tongue bulging at the back of the mouth. As a result, the acoustic characteristics of stopping are also present in backing, such as silence gap, burst, VOT, and noise. Figure 2 depicts the spectrogram difference between the backing and normal pronunciation.

#### 1.1.3. FCDP

**Figure 3:**
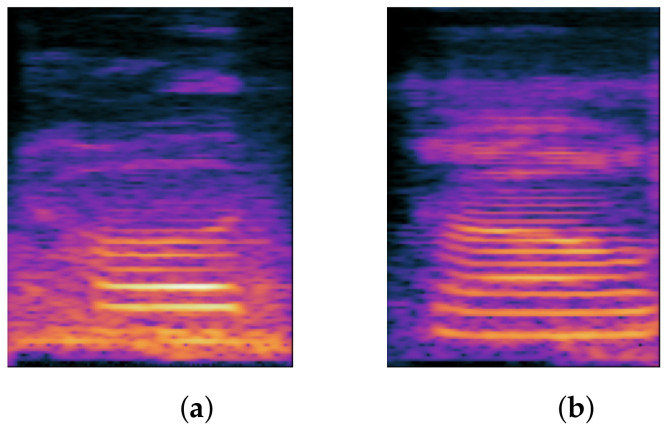
Spectrum comparison of FCDP and correct: (**a**) FCDP; (**b**) correct.

The final consonant is composed of a vowel and a coda and is pronounced by progressing from vowel to consonant. The final consonant is divided into two segments: the stop coda and the nasal coda. However, only the nasal coda contains the following consonants in the Chinese phonetic alphabet: ㄢ/an/, ㄣ/ǝn/, ㄤ/ɑŋ/, ㄥ/ɤŋ/. Therefore, the final consonant is considered as syllable structure component, and the deletion of the final consonant is referred to as the FCDP. The following section discusses the final consonant’s composition. The final consonant is categorized by the vowel ㄚ/ä/ or ㄜ/ɤ/, followed by /n/ or /ŋ/ at the end of the rhyme (coda), which can be roughly divided into two groups: ㄤ/ɑŋ/, ㄢ/an/ and ㄣ/ən/, ㄥ/ɤŋ/. When we pronounce ㄢ/an/, we place our tongue at its lowest point and slowly raise the tip of the tongue, allowing air to flow out of the nasal cavity; when we pronounce ㄤ/ɑŋ/, we also place our tongue at its lowest point and slightly open our mouth, allowing air to flow out of the nasal cavity while we keep our mouth open and pronounce the velar nasal /ŋ/. When pronouncing ㄣ/ən/ or ㄥ/ɤŋ/, the tongue is positioned in the mouth without moving up, down, forward, or backward, forming the vowel position of ㄜ/ɤ/, and the tongue tip moves up and out through the nasal cavity, producing an alveolar nasal /n/. To produce a response, on the other hand, a vowel position of ㄜ/ɤ/ is formed first; then the mouth remains open, and the airflows out of the nasal cavity, maintaining the mouth open and producing the velar nasal /ŋ/. Figure 3 depicts the spectrogram difference between the FCDP and normal pronunciation.

#### 1.1.4. Affrication

**Figure 4:**
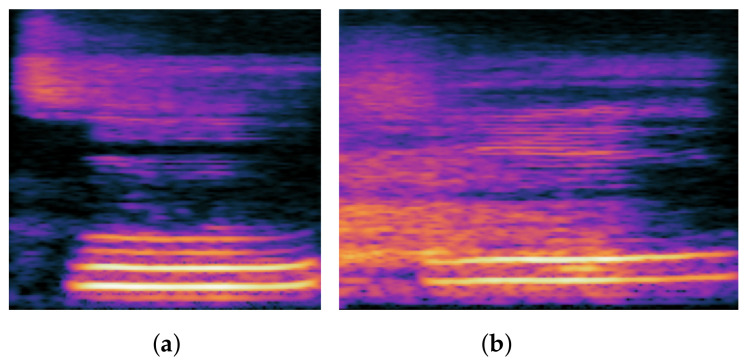
Spectrum comparison of affrication and correct: (**a**) affrication; (**b**) correct.

An affricate contains both stop and fricative features, so when it is pronounced, the oral constellation will first produce the stop feature and then the fricative feature. In Chinese pronunciation, there are six affricates: ㄗ/$\overset{⌢}{\mathrm{ts}}$/, ㄘ/ ${\overset{⌢}{ʈʂ}}^{h}$ /, ㄓ/$\overset{⌢}{ʈʂ}$/, ㄔ/${\overset{⌢}{\mathrm{ts}}}^{h}$/, ㄐ/$\overset{⌢}{tɕ}$/ and ㄑ/${\overset{⌢}{tɕ}}^{h}$/. When other phonemes are mispronounced as the six phonemes listed above, they become affrication. The so-called affricate is a closed tone that lasts for a period of time, forming a block and holding it. However, during the burst, the mouth does not completely release the airflow, or rather forms a small gap between the tongue and the hard palate, allowing the airflow to pass through the gap and produce a friction noise. When we examine the spectrogram, we can see that the affricate consonant has the acoustic characteristics of both the stop consonant and the fricative consonant, such as a silent period, a burst, and a short noise. However, the characteristics of the stop consonant are very dynamic, as they can change quickly and dramatically, and we can usually distinguish between them based on this characteristic. Figure 4 depicts the spectrogram difference between the affrication and normal pronunciation.

### 1.2. State of the Art

Despite the enormous potential demand for automatic SSDs classification, some scholars have also researched SSD in different languages. Anjos et al. [9] proposed identifying sibilant phonemes in European Portuguese using deep convolutional neural networks. According to [10], it identified six dental errors in Polish-speaking youngsters using a deep network. Hammami et al. [11] presented a method based on a real-world database of native Arabic-speaking children’s voice recordings. Based on the aforementioned research, it is evident that SSD classification using deep learning is feasible, although it is currently only used to identify and classify specific single consonants. On the other hand, relatively few studies have been conducted on Standard Chinese. There are two issues that make detecting the features of different construal errors challenging. First, when growing children attempt to pronounce constantly, the instability of co-constructive motions manifests. Second, the numerous features included in a single construal category are diverse, resulting in the difficulty of classification. Recent studies have classified and identified phonetic categories using deep learning architectures. Numerous model architectures are used, such as recurrent neural networks [12], convolutional neural networks [13], long short-term memory [14], and other deep learning frameworks. The model is fed a two-dimensional spectrogram or Mel-frequency cepstral coefficients (MFCC) data.

### 1.3. Aims and Scope

Our study aims to develop a reliable data analysis procedure for the computer-assisted diagnosis of SSDs in children. The goal is to provide a solution of detecting and classifying four types of speech sound errors in Mandarin Chinese. We collected a corpus of speech samples from 90 children aged 3 to 6, along with detailed diagnostic instructions provided by an SLP. The study is divided into three groups of experiments on pronunciation disorders. We train and compare our gathered dataset for speech sample categorization using three standard architectures: EfficientNet [15], DenseNet [16], and InceptionV3 [17]. We extract acoustic characteristics from sounds using a three-channel Mel-Spectrogram [18]. To aid the model’s learning when trained on custom datasets, we employ various data augmentation techniques [19] on our dataset.

### 1.4. Paper Structure

The following is the overall structure of this paper. Section 2 discusses the methods for pre-use treatment and model training. Section 3 provides a thorough description of the experimental findings. Section 4 discusses the potential reasons why different sound samples influence the accuracy and the bottleneck in the results. Section 5 concludes the work and future directions.

## 2. Materials and Methods

**Figure 5:**
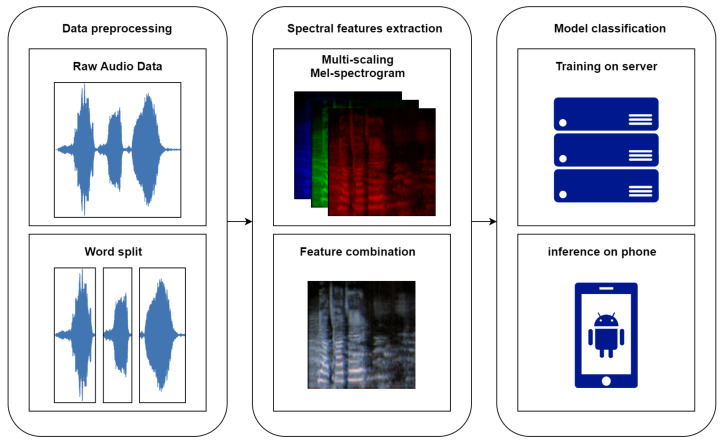
Overall workflow of the speech sound disorders classification.

The SSD classification task was carried out in accordance with the workflow depicted in Figure 5 and detailed in the following sections.

### 2.1. Collecting and Labeling Audio Samples

The study enrolled preschool children aged 3–6 years who had been diagnosed with speech and language impairment at a rehabilitation clinic or were referred by their kindergarten teachers as having a possible speech and language impairment. Between January and December 2021, a total of 90 children were enrolled, with the age and gender distributions shown in Table 1. We excluded cases with the following conditions: speech disorders caused by congenital central nerve injury (e.g., cerebral palsy); speech disorders caused by abnormal oral structures (e.g., cleft lip and palate); co-occurring intellectual disabilities; emotional behavior disorders (e.g., autism); speech disorders caused by hearing impairment; and family reluctance. Prior to the trial, the protocol was approved by the Cathay Hospital IRB. Consent was obtained verbally and in writing from the child’s parents or legal guardians to participate in the study.

**Figure 6:**
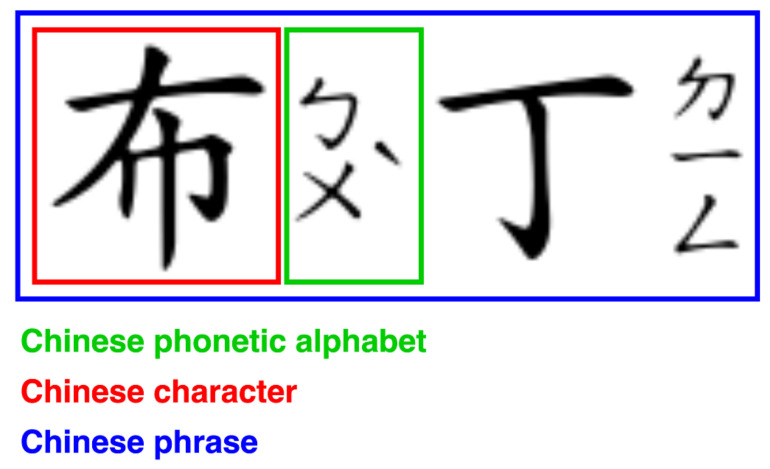
Definition of Chinese words.

Voice data were collected using a tablet computer with a microphone attached. We used rode’s smartLav microphone clipped to the subject’s clothing collar. For this task, we programmed an app to be installed on a Samsung Galaxy Tab S3 tablet. The microphone acquired the signal at a sampling frequency of 44.1 kHz and transmitted it to the tablet computer, and stored it in 16-bit depth. The database consists of 96 Chinese phrases, made up of 37 Chinese phonetic alphabets, each of which appears at the beginning, middle, and end of the word. The definition of Chinese words is shown in Figure 6. The Chinese phrases were illustrated with pictures, and the task for the child was to name the pictures spontaneously. For a detailed list of the Chinese phrases, please refer to Table A1. For each recording, two SLPs prepared diagnostic notes. The evaluation was performed to identify pathological pronunciation. In addition, abnormal intonation sounds were analyzed, and pathological types were annotated. Four types of articulation were collected:

- Stopping.
- Backing.
- FCDP.
- Affrication.

Before collecting the corpus, we expected a single model to identify the corresponding error category based on the phonetic sound of a single word. When compared to other languages, Chinese SSDs have more then 15 different error categories. Still, statistical analysis of the corpus we collected revealed that four or five of them are more common in clinical cases, implying that the other categories are relatively rare. Because it is challenging to train deep learning with extremely unbalanced or irregular data categories, we discussed with the SLPs. We determined that it would be better to start with the most common types in the clinical setting.

Two different kinds of speech samples were used: complete Chinese phrases and single Chinese characters. Each recorded speech sample contains a complete Chinese phrase, and a single Chinese character sample is extracted from each phrase sample. Following are the justifications for this action: The phrases are designed to use the 37 Chinese phonetic alphabets, arranging each phonetic symbol to appear in the front, middle, and back of common Chinese phrases. It is possible for patients to make SSDs in different positions when pronouncing a word or for various positions to contain different types of SSDs. In the case of the marker samples, only a single type of SSD is indicated in the SSD label of Chinese phrases. The marker data do not contain possible locations and multiple classes. To solve these problems, we designed Experiment 2 and recreated the dataset.

All samples were re-syllabified by acoustic experts, and two SLPs rigorously labeled all Chinese-single-character samples. To increase the accuracy and reliability of SLPs diagnostic results, only samples with consistent SLPs labels were preserved for subsequent studies. The SLPs labeling program used our custom-built labeling software to listen to each audio file and click on the error category option to which the sample belonged, after which the software generated a labeled file.

### 2.2. Data Pre-Processing

To preserve space and time information in the conversion of sound features, we chose Mel spectrograms as the feature representation. To perform transfer learning in a standard model pre-trained with image net, we used a three-channel Mel spectrogram. On each channel, the MelSpectrogram was calculated using various window widths and hop lengths of {25 ms, 10 ms}, {50 ms, 25 ms}, and {100 ms, 50 ms}. Different window widths and hop lengths guaranteed that each channel had varying amounts of frequency and temporal information.

To avoid overfitting during training and to make more efficient use of the limited sample, we used a variety of standard sound augmentation methods, as shown in the table below:

- Increase/decrease the pitch by two semitones.
- Shift the time by 10%.
- Scaling the speed by random number within ±25%.
- The input audio signal is compressed using dynamic range compression.
- Increase/decrease volume by a random number of decibels in [3, 3] dB.
- Random noise in the range [0, 10] dB is added (SNR).

All expansions were implemented using ffmpge [20] and python librosa packages [21]. After augmentation, we had nine times more data.

### 2.3. Models

We used three standard models to solve our problems. The following are the models:

1. EfficientNet [15]: They use neural architecture search to create a new baseline network and scale it up to create the EfficientNet family of models, which outperform previous ConvNets in accuracy and efficiency. EfficientNet uses a new scaling method that uses a simple but highly effective compound coefficient to scale all depth/width/resolution dimensions uniformly. EfficientNet shows how to scale up MobileNets and ResNets with this method.
2. DenseNet [16]: Dense Convolutional Network (DenseNet) is a feed-forward network that connects each layer to every other layer. The network has L(L + 1)/2 direct connections, whereas traditional convolutional networks with L layers have L connections between each and its subsequent layers. All previous layers’ feature maps are used as inputs into each layer, and their feature maps are used as inputs into all successive layers.
3. InceptionV3 [17]: The Inception architecture has been shown to achieve excellent performance while using a small amount of computational power. Inception network training is significantly accelerated when residual connections are used. By a razor-thin margin, residual Inception networks outperform similarly priced Inception networks without residual connections. They present several new streamlined Inception network architectures, both residual and non-residual.

The model’s trainable parameters and size are provided in Table 2.

### 2.4. Training Environments

Due to the dataset’s small sample size and data imbalance, we resolved the issue using class weights. We created a 5-folder cross-validation dataset for training and evaluating the model. We separated the data into training and validation at 80%, 20%, respectively. We configured the batch size to be 128, the number of epochs to 15, the training optimizer to be Adam, and the learning rate to 0.0001. Our loss function used categorical cross-entropy in Experiments 1 and 3 and binary cross-entropy in Experiment 2. The model with the lowest validation loss was saved as the result of each training session. Training and validation were carried out by a Keras-based TensorFlow platform (version 2.4) on Nvidia Tesla V100 with 32GB RAM. For training the same model, the same framework, hyperparameter settings, and training procedures were used.

### 2.5. Experiment Methods

#### 2.5.1. Experiment 1—Multi-Class Classification Using a Single Chinese Phrase

In this experiment, three standard models were used to predict four error categories by entering complete Chinese phrases. First, all audio files were labeled according to the category corresponding to the diagnostic label of SLPs. Then the feature map was processed to [128, 256, 3] size according to the preprocessing method in Section 2.3. Finally, five folders were created for cross-validation, and the number of data is shown in Table 3.

#### 2.5.2. Experiment 2—Binary Classification Using a Single Chinese Character

To mark the location of the misconstructions more precisely, the acoustic experts re-cut all Chinese phrases into individual sound files according to the Chinese characters. It means that each sample will contain only one Chinese character. The two SLPs re-evaluated the segmented samples and selected those with more significant error characteristics to produce a single-Chinese-character dataset. To evaluate the model’s ability to discriminate among the accurate samples, the new dataset with corresponding correctly pronounced samples was used to test the performance of the model for binary classification. Since the length of the sound sample becomes shorter after cutting, a feature size of [128, 128, 3] in the pre-processing is sufficient to include the sample features. In Experiment 2, the model took single-Chinese-character samples as input and output them as a correct category or incorrect category, and the amount of data is shown in Table 4 and Table 5.

#### 2.5.3. Experiment 3—Multi-Class Classification Using a Single Chinese Character

In Experiment 3, to further verify the ability of the model to discriminate the four types of errors in a single model, we repackaged the dataset from Experiment 2 to leave only the samples of error tones. The sample size of the dataset is shown in Table 6. In this experiment, the input of the model was a single Chinese character sample and the output was four error categories.

#### 2.5.4. Runtime of the Developed Application

We converted the trained models into TensorFlow Lite models (.tflite), and measured the inference time of all models on an Android mobile device. Given that the model is intended to be used in real-time by physicians or patients via smartphones, the time taken to infer is also critical. We used the performance measurement application [22] provided by the official Tensorflow website for performance evaluation. We tested all the models used using Google Pixel 6 with Android 12.

## 3. Results

To describe the experimental results, this chapter is divided into several subheadings. The first section will look into the efficacy of using Chinese phrases as classifier input. The second section investigates the effectiveness of Chinese characters in contrast. The third subsection investigates the efficacy of Chinese characters in the classifier. Furthermore, real-time inference on mobile devices is provided to demonstrate the viability of edge prediction. Initially, the unbalanced dataset led to ineffective training outcomes, which were not significantly enhanced until we implemented the balancing measures of class weights and data augmentation.

### 3.1. Experiment 1—Multi-Class Classification Using a Single Chinese Phrase

**Figure 7:**
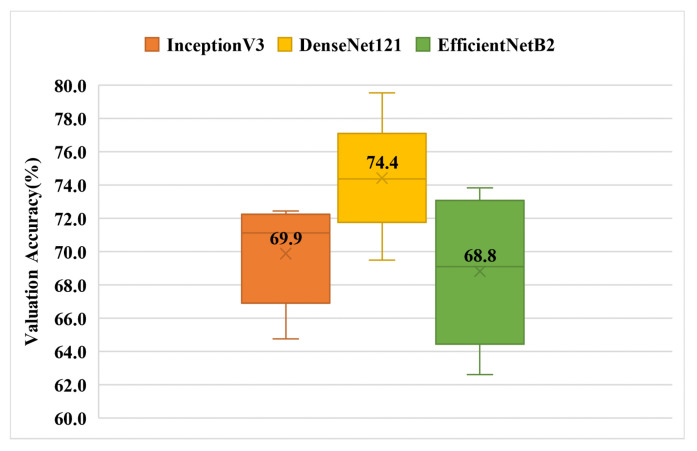
Validation accuracy of single Chinese phrase multi-category classification box plot (Experiment-1). The top and bottom of the box are the interquartile ranges (75th and 25th percentile) centered around the median value (50th percentile). The whiskers represent the minimum and maximum validation accuracy values. Table 8 presents the results in detail.

To verify the feasibility of the model for classifying SSDs, we performed cross-training on three standard models. Figure 7 shows the training results of the dataset on each model. The average cross-validation results for the three models were as follows: InceptionV3 with a result of 70%, DenseNet121 with a result of 74%, and EfficientNetB2 with a result of 69%. Table 7 shows the confusion matrix with the best accuracy among all the results.

**Table 7:** One of the most effective confusion matrices in Experiment 1 when the DenseNet121 model was used. Rows indicate output classes, columns indicate target classes.

	FCDP	Affrication	Backing	Stopping
FCDP	120	0	0	2
Affrication	4	65	0	4
Backing	7	2	20	8
Stopping	8	3	3	262

**Table 8:** Validation accuracy of single Chinese phrase multi-category classification.

Fold Number	InceptionV3	DenseNet121	EfficientNetB2
1	69.0	74.4	66.3
2	72.1	74.0	69.1
3	72.4	79.5	73.8
4	64.8	69.5	62.6
5	71.1	74.7	72.3
Average Value	69.9	74.4	68.8

### 3.2. Experiment 2—Binary Classification Using a Single Chinese Character

**Figure 8:**
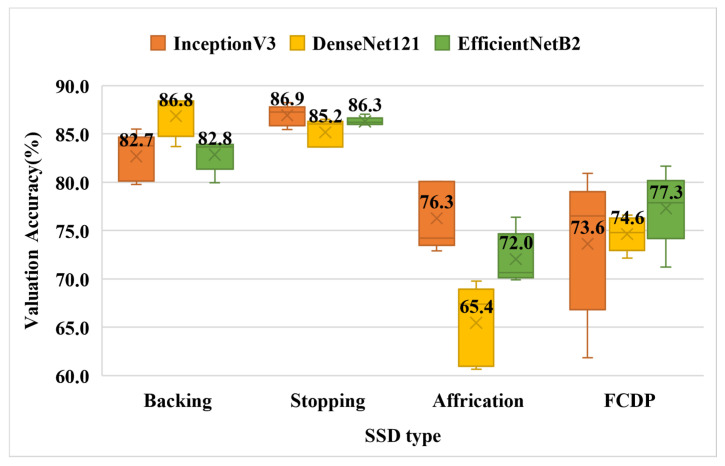
Validation accuracy of single Chinese character binary classification box plot (Experiment-2). Table 9, Table 10, Table 11 and Table 12 present the results in detail.

Figure 8 shows the accuracy results of the four types of binary classifications with phonetic errors on the three models. The displayed numbers are the average of the cross-validation results, and the following are the best results in each category: backing is 86.8 percent of DenseNet121; stopping is 86.9 percent of InceptionV3; Affricate is 76.3 percent of InceptionV3; and FCDP is 76 percent of EffcientNetB2. It can be found that the results of affrication and FCDP in each folder are relatively different, which we speculate is due to the fact that these two categories contain more Chinese characters, and the number of samples currently collected is not enough to satisfy the plurality of data.

**Table 9:** Validation accuracy of single Chinese character binary classification of backing class.

Fold Number	InceptionV3	DenseNet121	EfficientNetB2
1	83.8	88.4	79.9
2	79.8	83.7	83.9
3	83.8	85.8	84.0
4	80.5	88.4	82.8
5	85.5	88.0	83.7
Average Value	82.7	86.8	82.8

**Table 10:** Validation accuracy of single Chinese character multi-category classification of stopping class.

Fold Number	InceptionV3	DenseNet121	EfficientNetB2
1	87.3	86.5	86.2
2	88.2	86.1	86.0
3	87.4	83.7	86.3
4	85.4	86.0	86.0
5	86.3	83.7	87.1
Average Value	86.9	85.2	86.3

**Table 11:** Validation accuracy of single Chinese character multi-category classification of affrication class.

Fold Number	InceptionV3	DenseNet121	EfficientNetB2
1	80.1	69.8	69.9
2	74.0	60.7	70.7
3	74.2	67.4	72.9
4	72.9	61.3	76.4
5	80.1	68.1	70.4
Average Value	76.3	65.4	72.0

**Table 12:** Validation accuracy of single Chinese character multi-category classification of FCDP class.

Fold Number	InceptionV3	DenseNet121	EfficientNetB2
1	71.8	74.8	77.9
2	76.5	76.6	77.1
3	61.8	73.7	81.7
4	80.9	72.2	78.6
5	77.1	76.0	71.2
Average Value	73.6	74.6	77.3

### 3.3. Experiment 3—Multi-Class Classification Using a Single Chinese Character

**Figure 9:**
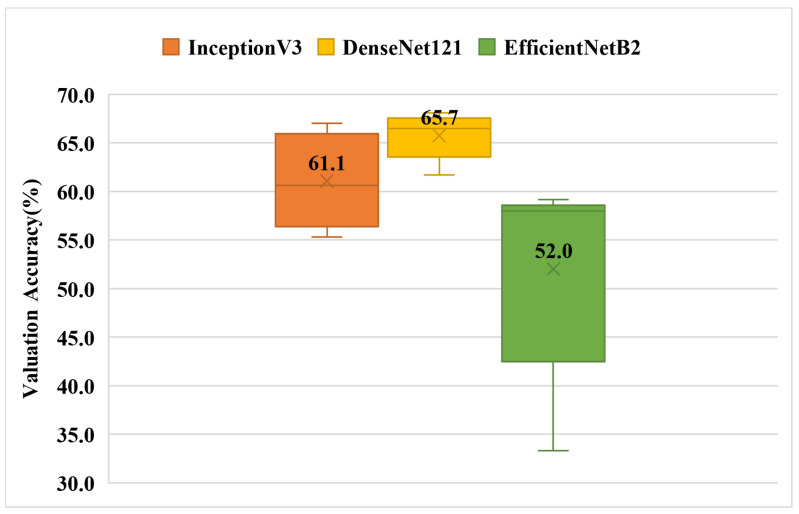
Validation accuracy of single Chinese character multi-category classification box plot (Experiment-3). Table 14 presents the results in detail.

The experimental results are shown in Figure 9. It can be found that the overall accuracy of the model has decreased somewhat compared with that of Experiment 1, but the confusion matrix in Table 13 shows that the model still has a certain level of discriminatory ability.

**Table 13:** One of the most effective confusion matrices in Experiment 3 when the InceptionV3 model was used. Rows indicate output classes, columns indicate target classes.

	Backing	Stopping	Affrication	FCDP
Backing	19	3	5	5
Stopping	7	27	11	3
Affrication	1	10	65	2
FCDP	2	0	7	22

**Table 14:** Validation accuracy of single Chinese character multi-category classification.

Fold Number	InceptionV3	DenseNet121	EfficientNetB2
1	55.3	65.4	51.6
2	60.6	61.7	58.0
3	64.9	66.5	58.0
4	57.5	67.0	59.2
5	67.0	68.1	33.3
Average Value	61.1	65.7	52.0

### 3.4. Runtime of the Developed Application

The performance of all the models is summarized in Table 15, and it is clear that the models we used meet the requirements for real-world usage scenarios. In other words, it only takes about three seconds for the GPU to predict all 96 Chinese phrases on the phone. The accuracy of the TFLite model run on a cell phone was nearly identical (less than one percent) to that of the original model run on a computer.

## 4. Discussion

The research presented in this article aims to develop a tool for analyzing SSD error classes based on deep learning. A workflow has been created to collect and train a model that categorizes SSDs. SLPs, who will be the primary beneficiaries, were involved in every aspect of the study. Experts tagged the data, then analyzed and experimented with it to train the model to detect and classify SSDs. The system is designed to help preschool children because diagnosis and intervention are most beneficial at this age.

The results show that the use of Chinese phrase samples for the current dataset is more effective than single Chinese character samples for model training. In general, the four types of error categories using either Chinese phrases or single Chinese characters can achieve good results in the current mainstream image classification neural networks. However, using Chinese phrase samples as model input is easier to train than single Chinese characters samples, contrary to the original expectation. Before the experiment, we hypothesized that reducing the range of speech marks would make it easier for the model to distinguish SSD classes.

Several factors may account for this, including the re-screening of all samples in the Chinese single-character dataset and the elimination of ambiguous or imprecise phonetic samples by SLPs. This reduced the number of samples in the dataset. Another possible reason is that the reduced sample length also means that the model cannot find the position of the Chinese character in the original vocabulary and the combination or variation with the preceding and following sounds. This may require further refinement of the tagging method and model design to verify whether the Chinese phrase or the Chinese character is more suitable for the composition of the SSDs classification input.

Experiments reveal that when all three models are trained under the identical conditions, the best achievable accuracy is comparable. However, the disparity between individual cross-training results is enormous. We believe this may have something to do with the size of the dataset. The corpus that we have compiled must continue to be expanded so that the model can completely learn the diversity of data and more specifics during the learning process. Based on the existing training environment, all three models can effectively train usable outcomes, but if we wish to further enhance the accuracy, we must increase the size of the voice samples.

## 5. Conclusions

In this paper, we investigated the idea of using neural networks to classify SSD categories in both binary and multi-category classifications. The task is to identify the error category by the pronunciation of common Chinese words. The categories include stopping, backing, FCDP, and affrication. With the progressive development of multi-dimensional CNN models, we used several standard models which are well established and powerful in image classification tasks for identification and classification. We used multi-dimensional spectral signals as input to the model, and the input features are composed of three two-dimensional Mel-Spectrogram feature maps.

We were able to classify four common types of SSD errors using monosyllabic speech samples and neural network models. This study is the first in Taiwan to apply deep learning to the treatment of SSDs, and its findings are based on the four most common articulation errors in Taiwan. Possibly in the near future, machine learning will be able to aid SLP and the patient’s treatment process. We found that with sufficient data, the neural network model is able to identify subtle differences in the characteristics of different prosodic errors in single Chinese characters. Other rare categories, in theory, can be successfully identified if sufficient samples of speech sounds are collected.

**Figure A1:**
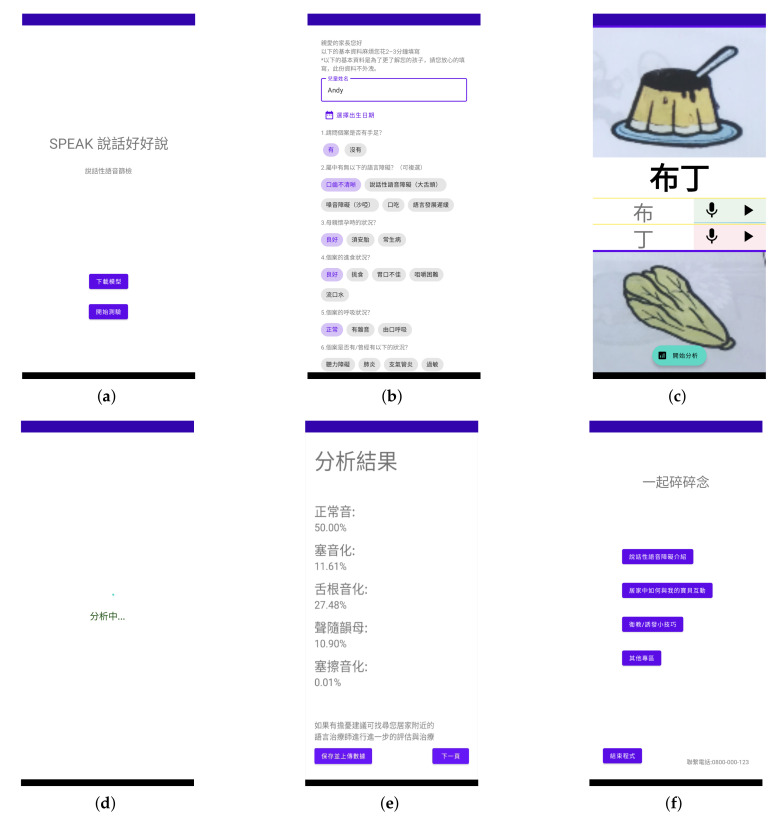
Screenshot of the mobile application: (**a**) Application main page: Users have the option of initiating a test or downloading the most recent classification model.; (**b**) User questionnaire page: The first step in the test procedure is to complete the questionnaire, which is primarily used for SSD background checks, such as whether the vocal organs are normal, etc.; (**c**) Recording interface: The recording session is the following step. The user can record by clicking the microphone button or play the sound file by clicking the play button.; (**d**) Analysis page: There are pages awaiting analysis.; (**e**) Result report page: Results page gives results for different SSDs categories.; (**f**) Health information link page: Links for Health Education and Promotion.

Currently, we are converting the trained models into models that can be predicted on smartphones in a timely manner through tensorflow lite. The pre-developed app provides a complete experience of real-time recording and analysis and is being clinically tested in the rehabilitation department of a hospital. The demo of the application is shown in Figure A1. The current accuracy of 86% is sufficient for rapid screening for parents prior to medical treatment or self-assessment for long-term review and correction. This will save many patients or SLPs a lot of time.

## Figures and Tables

**Table 1:** Distribution of subjects’ ages and genders.

Age	Sex		Total
	Female	Male	
3	8	14	22
4	11	18	29
5	11	20	31
6	4	4	8
Total	34	56	90

**Table 2:** Trainable parameters and size of the model.

Experiment	Model	Trainable Params	Size (mb)
e1	DenseNet121	6,957,956	82
	EfficientNetB2	7,706,630	91
	InceptionV3	21,776,548	251
e2	DenseNet121	6,955,906	82
	EfficientNetB2	7,703,812	91
	InceptionV3	21,772,450	251
e3	DenseNet121	6,957,956	82
	EfficientNetB2	7,706,630	91
	InceptionV3	21,776,548	251

**Table 3:** The amount of data on single-Chinese-phrase dataset. Training segment contains augmented data.

CV	Training Segments				Test Segments			
	FCDP	Affrication	Backing	Stopping	FCDP	Affrication	Backing	Stopping
Fold1	4401	2628	1332	9936	122	72	37	276
Fold2	4401	2619	1332	9936	122	73	37	276
Fold3	4401	2619	1332	9936	122	73	37	276
Fold4	4401	2619	1332	9936	122	73	37	276
Fold5	4401	2619	1332	9936	123	73	37	276

**Table 4:** The amount of data on the single-Chinese-character dataset for training.

CV	Backing		Stopping		Affrication		FCDP	
	Incorrect	Correct	Incorrect	Correct	Incorrect	Correct	Incorrect	Correct
Fold1	1125	5724	1728	11,655	2853	5283	1089	3636
Fold2	1125	5724	1728	11,655	2853	5283	1089	3636
Fold3	1125	5724	1728	11,655	2853	5283	1089	3636
Fold4	1125	5724	1728	11,655	2853	5283	1089	3636
Fold5	1116	5715	1728	11,646	2844	5274	1080	3636

**Table 5:** The amount of data on the single-Chinese-character dataset for validation.

CV	Backing		Stopping		Affrication		FCDP	
	Incorrect	Correct	Incorrect	Correct	Incorrect	Correct	Incorrect	Correct
Fold1	31	158	48	323	79	146	30	101
Fold2	31	159	48	324	79	147	30	101
Fold3	31	159	48	323	79	146	30	101
Fold4	31	159	48	324	79	147	30	101
Fold5	32	159	48	324	80	147	31	101

**Table 6:** The amount of data on single-Chinese-character dataset.

CV	Training Segments				Test Segments			
	Backing	Stopping	Affrication	FCDP	Backing	Stopping	Affrication	FCDP
Fold1	1125	1728	2853	1089	31	48	79	30
Fold2	1125	1728	2853	1089	31	48	79	30
Fold3	1125	1728	2853	1089	31	48	79	30
Fold4	1125	1728	2853	1089	31	48	79	30
Fold5	1116	1728	2844	1080	32	48	80	31

**Table 15:** The performance values below are measured on Android 12 from Google Pixel 6.

Experiment	Model Name	CPU	GPU	Model Size (MB)
e1	DenseNet 121	489 ms	32 ms	27
	EfficientNet B2	438 ms	-	29
	Inception V3	399 ms	35 ms	83
e2	DenseNet 121	231 ms	29 ms	27
	EfficientNet B2	241 ms	-	29
	Inception V3	170 ms	36 ms	83
e3	DenseNet 121	236 ms	30 ms	27
	EfficientNet B2	235 ms	-	29
	Inception V3	171 ms	36 ms	83

## Data Availability

Not applicable.

## Abbreviations

The following abbreviations are used in this manuscript:

SSDs	Speech sound disorders
SLPs	Speech-language pathologists
MFCC	Mel-frequency cepstral coefficients
FCDP	Final consonant deletion process

## Appendix A

Table A1 shows the list of Chinese phrases collected in this experiment. Each participant’s pronunciation sample was recorded according to the phrases in the list. On average, it took about 30 min to record one participant. The length of each Chinese phrase was limited to less than three seconds.

**Table A1:** Chinese phrase list.

Chinese Phease	IPA	Translation in English	Chinese Phease	IPA	Translation in English
布丁	Bùdīng	pudding	閃電	shǎndiàn	lightning
麵包	miànbāo	bread	牙刷	yáshuā	toothbrush
大白菜	dàbáicài	Chinese cabbage	直升機	zhíshēngjī	helicopter
螃蟹	pángxiè	Crab	日歷	rìlì	calendar
奶瓶	nǎipíng	baby bottle	超人	chāorén	superman
蓮蓬頭	liánpengtóu	shower head	大榕樹	dàróngshù	Large banyan
帽子	màozi	hat	走路	zǒulù	walk
玉米	yùmǐ	corn	洗澡	xǐzǎo	bath
捉迷藏	zhuōmícáng	hide and seek	水族箱	shuǐzúxiāng	aquarium
鳳梨	fènglí	pineapple	草莓	cǎoméi	Strawberry
衣服	yīfú	clothing	洋蔥	yángcōng	onion
吹風機	chuīfēngjī	hair dryer	上廁所	shàngcèsuǒ	To the restroom
動物	dòngwù	animal	掃把	sàobǎ	broom
蝴蝶	húdié	Butterfly	垃圾	lèsè	Rubbish
看電視	kàndiànshì	watch TV	去散步	qùsànbù	go for a walk
太陽	tàiyáng	Sun	衣服	yīfú	clothing
枕頭	zhěntou	Pillow	果醬	guǒjiàng	jam
一條魚	yītiáoyú	a fish	指甲刀	zhǐjiǎdāo	nail clippers
鈕扣	niǔkòu	button	筷子	kuàizi	Chopsticks
電腦	diànnǎo	computer	烏龜	wūguī	tortoise
喝奶昔	hēnǎixī	drink milkshake	去公園	qùgōngyuán	go to the park
老虎	lǎohǔ	Tiger	杜鵑花	dùjuānhuā	Rhododendron
恐龍	kǒnglóng	Dinosaur	選擇	xuǎnzé	choose
養樂多	yǎnglèduō	Yakult	缺點	quēdiǎn	shortcoming
果凍	guǒdòng	jelly	太陽	tàiyáng	Sun
烏龜	wūguī	tortoise	大海	dàhǎi	the sea
去公園	qùgōngyuán	go to the park	喝奶昔	hēnǎixī	drink milkshake
筷子	kuàizi	Chopsticks	草莓	cǎoméi	Strawberry
貝殼	bèiké	shell	貝殼	bèiké	shell
巧克力	qiǎokèlì	chocolate	水族箱	shuǐzúxiāng	aquarium
漢堡	hànbǎo	hamburger	帽子	màozi	hat
大海	dàhǎi	the sea	麵包	miànbāo	bread
救護車	jiùhùchē	ambulance	一條魚	yītiáoyú	a fish
膠帶	jiāodài	adhesive tape	鈕扣	niǔkòu	button
果醬	guǒjiàng	jam	枕頭	zhěntou	Pillow
指甲刀	zhǐjiǎdāo	nail clippers	中秋節	zhōngqiūjié	Mid-Autumn Festival
鉛筆	qiānbǐ	pencil	漢堡	hànbǎo	hamburger
鋼琴	gāngqín	piano	電腦	diànnǎo	computer
中秋節	zhōngqiūjié	Mid-Autumn Festival	看電視	kàndiànshì	watch TV
信封	xìnfēng	envelope	信封	xìnfēng	envelope
點心	diǎnxīn	dessert	鋼琴	gāngqín	piano
口香糖	kǒuxiāngtáng	chewing gum	吃點心	chīdiǎnxīn	eat dessert
站牌	zhànpái	stop sign	螃蟹	pángxiè	Crab
蠟燭	làzhú	Candle	果醬	guǒjiàng	jam
擦桌子	cāzhuōzi	wipe the table	口香糖	kǒuxiāngtáng	chewing gum
抽屜	chōutì	drawer	鳳梨	fènglí	pineapple
警察	jǐngchá	Policemen	奶瓶	nǎipíng	baby bottle
柳橙汁	liǔchéngzhī	orange juice	蓮蓬頭	liánpengtóu	shower head

## Appendix B

The following Figure A1 shows the final mobile application, which can be used by users to perform real-time testing of the SSD category on their cell phones. The application is divided into three main functions. First, users can download the latest version of the SSD identification model from the first screen. Then, after filling out the basic questionnaire, the user can enter the Chinese phrase recording stage, and after each phrase is recorded, the user can listen to it again and again to confirm that it is completely recorded. The program will calculate the prediction results for each phrase and present them to the user in a report. Users can also choose whether or not to provide the recording data for backend analysis and addition to the dataset.
